# Invert emulsion: Method of preparation and application as proper formulation of entomopathogenic fungi

**DOI:** 10.1016/j.mex.2016.02.001

**Published:** 2016-02-21

**Authors:** Yacoub A. Batta

**Affiliations:** Laboratory of Plant Protection, Department of Plant Production & Protection, Faculty of Agriculture & Veterinary Medicine, An-Najah National University, Nablus, West Bank, Palestine

**Keywords:** Description of method of preparing and applying invert emulsion as proper formulation of entomopathogenic fungi, Technique of preparation, Method of application, Invert emulsion, Formulation, Entomopathogenic fungi, Targeted insects

## Abstract

The present article describes the technique used for preparing the invert emulsion (water-in-oil type) then, selecting the most proper formulation of invert emulsion for being used as a carrier formulation of entomopathogenic fungi. It also describes the method used for testing the efficacy of the formulated fungi as biocontrol agents of targeted insects. Detailed examples demonstrating the efficacy of formulated strains of entomopathogenic fungi against certain species of insect pests were included in the present article. The techniques and methods described in this article are reproducible and helpful in enhancing the effectiveness of formulated fungi against wide range of targeted insects in comparison with the unformulated form of these fungi. Also, these techniques and methods can be used effectively in crop protection and in the integrated pest management programs. Finally, it is important to indicate that the ingredients used for preparation of the invert emulsion have no environmental side-effects or health risks since these ingredients are safe to use and can be used in manufacturing of cosmetics or as food additives.

• Description of method used for preparation of invert emulsion (water-in-oil type) and selecting the most stable and non-viscous emulsion.
• Description of technique used for introducing the entomopathogenic fungi into the selected stable and non-viscous invert emulsion.
• Description of method for testing the efficacy of introduced entomopathogenic fungus into the selected invert emulsion against targeted insects with detailed examples on the efficacy testing.

## Graphical abstract

**Figure:**
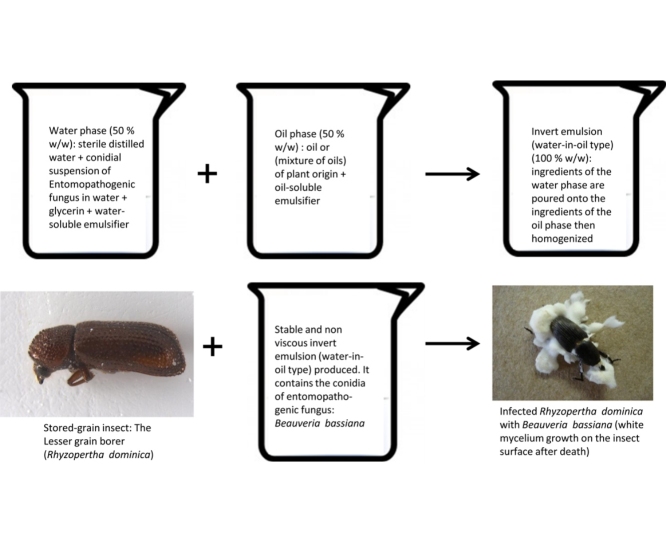

## Method details

The objectives of the present article are: (i) to describe the technique used for production of stable and non-viscous invert emulsion using new ingredients and proper proportions during the preparation; (ii) to describe the method used for introducing the selected strains of entomopathogenic fungi into the formulation of invert emulsion characterized by the highest stability and lowest viscosity; (iii) to describe the method used for testing the efficacy of formulated strains of entomopathogenic fungi in invert emulsion against the targeted insect pests; (iv) to demonstrate the effectiveness of formulated strains of entomopathogenic fungi in invert emulsion by giving detailed examples on biocontrol of targeted insect pests from our previous research.

### Technique of preparation and selection of proper formulation of invert emulsion

Because the type and proportion of ingredients entering in invert emulsion can be changed according to the stability and viscosity of emulsion produced, each combination of these ingredients that could be obtained after preparation is considered a new formulation. Each combination is consisted of two phases: (i) water or aqueous phase comprising of sterile distilled water, glycerine and water-soluble emulsifier, and (ii) oil phase comprising of oil (preferably of plant-origin) and oil-soluble emulsifier. The ingredients of each phase are prepared separately by combining them on a weight basis then, the two phases are mixed together at a high speed (20,000–25,000 rpm for 1.5 min using a homogenizer). The high speed of mixing is necessary to ensure the homogeneity of the emulsion produced and to obtain longer stability. The proportion of each phase in the final invert emulsion is fixed (50%, w/w) to make sure that enough quantity of water is available in the final emulsion. However, the percentage of the above-mentioned ingredients in each phase before mixing is not fixed but can be modified during preparation of each combination in order to have a more stable and less viscous emulsion. It is thus recommended to prepare large number of combinations then choose the most proper combination which is characterized by having the highest stability and lowest viscosity. Therefore, the selected formulation of invert emulsion should be the combination which has a high stability and low viscosity. For example, in Table 1 that comprises of 6 combinations of invert emulsion, the combination abbreviated “IE # 4” was selected as the most proper formulation of invert emulsion because it has shown a high stability for long time with no viscosity, therefore this combination has been chosen as a proper formulation of invert emulsion for introducing the selected strains of entomopathogenic fungi. The ingredients of the selected formulation “IE # 4” are: a mixture of two oils of plant-origin (soybean oil: 28.50%, w/w and coconut oil: 19.50%, w/w), oil-soluble emulsifier (Tween 20: 2.0%, w/w), sterile distilled water (45.00%, w/w), glycerine (4.25%, w/w), and water-soluble emulsifier (Dehymuls k: 0.75%, w/w).

### Method of introducing the selected strains of entomopathogenic fungi into the selected formulation of invert emulsion

The introduction of the selected strains of entomopathogenic fungi into the most proper formulation of invert emulsion is done at first into the water or aqueous phase of the emulsion which has finally become consisted of the following ingredients: sterile distilled water, conidial suspension of the selected entomopathogenic fungus in water, glycerine, and water-soluble emulsifier. The ingredients of oil phase are not changed and remain as they are indicated in the above section. The ratio of these two phases in the final invert emulsion after introduction of entomopathogenic fungi was the same as indicated above (50%, w/w). The concentration of the conidial suspension of the selected strain of entomopathogenic fungi in the formulation should be enough and not less than 1 million conidia per ml. Mixing of the two phases after introduction of the conidial suspension of the fungus into the water phase is done as indicated in the previous section to ensure the homogeneity and stability of the emulsion produced.

### Method of testing the efficacy of formulated strain of entomopathogenic fungi after introduction into the selected formulation of invert emulsion

The efficacy of formulated strain of entomopathogenic fungi in invert emulsion was tested against the targeted insects according to the following method: (i) application of the selected formulation of invert emulsion containing the strain of entomopathogenic fungi to the targeted insects directly or to the bottom of each container into which the insects will be transferred. This application is performed by spaying the formulation using a small calibrated hand sprayer equipped with a nozzle suited to low volume spray application, (ii) The same spray volume is applied in the control treatment with Blank formulation of invert emulsion or with sterile distilled water, (iii) introduction of insects (10 male and female adults or larvae) of each species of targeted insects into each container, then covered with a piece of cheese-cloth mesh fastened by a rubber band for ventilation, each container or group of insects represents a treatment replicate and 4 containers or groups of insects representing 4 replicates are used, (iv) incubation of containers or groups of insects is done at 25 ± 1 °C for 7–10 days before the assessment of insect mortality due to infection with the formulated strain of entomopathogenic fungus, (v) the experimental treatments used during each bioassay are: formulated strain of entomopathogenic fungus in invert emulsion, unformulated strain of entomopathogenic fungus (conidial suspension of the fungus in water), blank formulation of invert emulsion and the control (untreated insects with entomopathogenic fungus), (vi) evaluation of the treatment effect of formulated strain of entomopathogenic fungi is done according to the assessment of mortality in the treated insects compared to the control and other treatments. The assessment is done by calculating the mean % of insects mortality due to fungus infection for comparison of the efficacy. Statistical analyses using ANOVA and Duncan's Multiple Range Test or Tukey's HSD test are used for these comparisons.

### Examples demonstrating the efficacy of formulated strains of entomopathogenic fungi in invert emulsion

Different formulations of invert emulsions containing strains of entomopathogenic fungi were prepared and then tested against different species of targeted insects during our previous research. The following examples demonstrate the effectiveness and reproducibility of techniques used for preparation and application of the invert emulsions as a proper formulation of entomopathogenic fungi:

***Example 1***: Strain MAPAL2 of the entomopathogenic fungus *Metarhizium anisopliae* was formulated in invert emulsion formulation and then tested against three stored grain insects (*Sitophilus granarius*, *Sitophilus oryzae* and *Rhyzopertha dominica*) (Table 2). The selected formulation of invert emulsion used to formulate the fungus strain has the following ingredients: (i) oil phase comprising of: 1 – mixture of two oils of plant-origin (soybean oil: 28.50%, w/w and coconut oil: 19.50%, w/w) characterized by having a high content of monounsaturated fat required for preparing a stable invert emulsion, 2 – oil-soluble emulsifier (Tween 20: 2.0%, w/w); (ii) water or aqueous phase comprising of: 1 – sterile distilled water (22.5%, w/w), 2 – conidial suspension of the entomopathogenic fungus in water (*M. anisopliae*, strain MAPAL2, 22.5% (w/w), concentration: 1 × 10^7^ conidia/ml), 3 – glycerine (4.25%, w/w), and 4 – water-soluble emulsifier (Dehymuls k: 0.75%, w/w). The total % of ingredients involved in preparation of invert emulsion was 100 (w/w). It is equally divided between the water phase and the oil phase (50:50%, w/w).

The treatment with the formulated strain (MAPAL2 of *M. anisopliae*) has caused the highest % of mean adult mortality of insects per strain of entomopathogenic fungi in comparison with the control (80.25% versus 8.83%, respectively, Table 2). Significant differences (at *P* = 0.05) were obtained between the average mean % of adult mortality of insects/strain of entomopathogenic fungi for the formulated, unformulated MAPAL2 strain, blank formulation of invert emulsion and the untreated control (80.25, 57.50, 18.83 and 8.83%, respectively, Table 2). Therefore, the formulation of the above strain in invert emulsion has enhanced its efficacy in comparison with the unformulated form since the average mean % of adult mortality of insects/strain of entomopathogenic fungi increased significantly from 57.50 to 80.25% (Table 2).

***Example 2***: Strain BBPAL1 of the entomopathogenic fungus *Beauveria bassiana* was formulated in invert emulsion formulation and then tested against three stored grain insects (*Sitophilus granarius*, *Sitophilus oryzae* and *Rhyzopertha dominica*) (Table 3). The selected formulation of invert emulsion used to formulate the fungus strain has the following ingredients: (i) oil phase comprising of: 1 – mixture of two oils of plant-origin (soybean oil: 28.50%, w/w and coconut oil: 19.50%, w/w) characterized by having a high content of monounsaturated fat required for preparing a stable invert emulsion, 2 – oil-soluble emulsifier (Tween 20: 2.0%, w/w); (ii) water or aqueous phase comprising of: 1 – sterile distilled water (22.5%, w/w), 2 – conidial suspension of the selected entomopathogenic fungus in water (*B. bassiana*, strain BBPAL1, 22.5% (w/w), concentration: 1 × 10^7^ conidia/ml), 3 – glycerine (4.25%, w/w), and 4 – water-soluble emulsifier (Dehymuls k: 0.75%, w/w). The total % of ingredients involved in preparation of invert emulsion was 100 (w/w). It is equally divided between the water phase and the oil phase (50:50% w/w).

The treatment with the formulated strain (BBPAL1 of *B. bassiana*) has caused the highest % of mean adult mortality of insects per strain of entomopathogenic fungi in comparison with the control (86.50% versus 9.08%, respectively, Table 3). Significant differences (at *P* = 0.05) were obtained between the average mean % of adult mortality of insects/strain of entomopathogenic fungi for the formulated, unformulated BBPAL1 strain, blank formulation of invert emulsion and the untreated control (86.50, 62.16, 17.58 and 9.08%, respectively, Table 3). Therefore, the formulation of the above strain in invert emulsion has enhanced its efficacy in comparison with the unformulated form since the average mean % of adult mortality of insects/strain of entomopathogenic fungi increased significantly from 62.16 to 86.50% (Table 3).

***Example 3***: Strain 149 of the entomopathogenic fungus *Beauveria bassiana* was formulated in invert emulsion formulation and then tested against the adults of the almond bark beetle (*Scolytus amygdali*) (Table 4). The selected formulation of invert emulsion used to formulate the fungus strains has the following ingredients: (i) oil phase comprising of: 1 – mixture of two oils of plant-origin (coconut oil: 19.00%, w/w + soybean oil: 28.50%, w/w), 2 – oil-soluble emulsifier (Tween 20: 2.5%, w/w); (ii) water or aqueous phase comprising of: 1 – sterile distilled water (20.25%, w/w), 2 – conidial suspension of the entomopathogenic fungus in water (*B. bassiana*, strain 149, 25.00% (w/w), concentration: 15.5 × 10^7^ conidia/ml), 3 – glycerine (4.00%, w/w), and 4 – water-soluble emulsifier (Dehymuls k: 0.75%, w/w). The total % of ingredients involved in preparation of invert emulsion was 100 (w/w). It is equally divided between the water phase and the oil phase (50:50%, w/w).

The treatment with the formulated strain (149 of *B. bassiana*) has caused the highest % of mean adult mortality of *S. amygdali* in comparison with the control (43.3 and 100% versus 0 and 6.7% for 4 and 10 days of treatment with the fungus, respectively, Table 4). Significant differences (at *P* = 0.05) were obtained between the mean % of adult mortality of insects for the formulated and unformulated *B. bassiana* strain (149) (43.3 and 100% versus 16.7 and 60.0% for 4 and 10 days of treatment, respectively, Table 4). Therefore, the formulation of the above strain in invert emulsion has enhanced its efficacy in comparison with the unformulated form since the mean % of adult mortality of insects increased significantly from 16.7 to 43.3%, respectively for 4 days after the treatment, and from 60.0 to 100%, respectively for 10 days after the treatment, (Table 4). Also, significant differences were obtained between the mean % of adult mortality of these treatments versus blank formulation of invert emulsion and the untreated control (43.3, 16.7 versus 10.0 and 0%, respectively for 4 days after the treatment and 100, 60.0 versus 16.7 and 6.7%, respectively for 10 days after the treatment, Table 4).

***Example 4***: Strain Meta 1 of the entomopathogenic fungus *Metarhizium anisopliae* was formulated in invert emulsion formulation and then tested against the adults of the lesser grain borers (*Rhyzopertha dominica*) (Table 5). The selected formulation of invert emulsion used to formulate the fungus strain has the following ingredients: (i) oil phase comprising of: 1 – mixture of two oils of plant-origin (soybean oil: 28.50%, w/w and coconut oil: 19.00%, w/w), 2 – oil-soluble emulsifier (Tween 20: 2.50%, w/w); (ii) water or aqueous phase comprising of: 1 – sterile distilled water (21.00%, w/w), 2 – conidial suspension of the entomopathogenic fungus in water (*M. anisopliae*, strain Meta 1, 24.25% (w/w), concentration: 6.5 × 10^8^ conidia/ml), 3 – glycerine (4.00%, w/w), and 4 – water-soluble emulsifier (Dehymuls k: 0.75%, w/w). The total % of ingredients involved in preparation of invert emulsion was 100 (w/w). It is equally divided between the water phase and the oil phase (50:50%, w/w).

The treatment with the formulated strain (Meta 1 of *M. anisopliae*) has caused the highest % of mean adult mortality of *R. dominica* in comparison with the control (92.0 versus 13.3%, respectively, Table 5). Significant differences (at *P* = 0.05) were obtained between the mean % of adult mortality of insects for the formulated and unformulated Meta 1 strain, blank formulation of invert emulsion and the untreated control (92.0, 53.3, 25.3 and 13.3%, respectively, Table 5). Therefore, the formulation of the above strain in invert emulsion has enhanced its efficacy in comparison with the unformulated form since the mean % of adult mortality of insects increased significantly from 53.3 to 92.0%, respectively (Table 5). Also, significant differences were obtained between the mean % of adult mortality of these treatments versus blank formulation of invert emulsion and the untreated control (92.0 and 53.3 versus 25.3 and 13.3%, respectively, Table 5).

***Example 5***: The Strain BG 1 of the entomopathogenic fungus *Beauveria bassiana* was formulated in invert emulsion formulation and then tested against the larvae of yellow mealworm (*Tenebrio molitor*) (Table 6). The selected formulation of invert emulsion used to formulate the fungus strain has the following ingredients: (i) oil phase comprising of: 1 – oil of plant-origin (Canola oil: 480 g/kg), 2 – oil-soluble emulsifier (Tween 20: 20 g/kg); (ii) water or aqueous phase comprising of: 1 – sterile distilled water (200 g/kg), 2 – conidial suspension of the entomopathogenic fungus in water (*B. bassiana*, strain BG 1, 250 g/kg, concentration: 1 × 10^8^ conidia/ml), 3 – glycerine (42.5 g/kg), and 4 – water-soluble emulsifier (Dehymuls k: 7.5 g/kg). The total weight of ingredients involved in preparation of invert emulsion was 1 kg. It is equally divided between the water phase and the oil phase of the emulsion (500 g: 500 g).

The treatment with the formulated strain (BG 1 of *B. bassiana*) has caused the highest % of mean larval mortality of *T. molitor* in comparison with the control (96.6% versus 0%, respectively, Table 6). Significant differences (at *P* = 0.05) were obtained between the mean % of larval mortality for the formulated, unformulated BG 1 strain, blank formulation of invert emulsion and the untreated control (96.6, 40.0, 23.3 and 0%, respectively, Table 6). Therefore, the formulation of the above strain in invert emulsion has enhanced its efficacy in comparison with the unformulated form since the mean % of larval mortality increased significantly from 40.0 to 96.6% (Table 6). However, no significant differences were obtained between the mean % of larval mortality for the unformulated *B. bassiana* strain and the blank formulation of invert emulsion (40.0 versus 23.3, respectively, Table 6).

## Conclusions

1. The technique used for preparation and selection of new formulations of invert emulsion (water-in-oil type) is optimized and described in the present article.
2. The selected formulations of invert emulsion should be characterized by having long stability and low viscosity.
3. The method of introducing entomopathogenic fungi into the selected formulation of invert emulsion is adjusted and described in the present article.
4. The formulated strains of entomopathogenic fungi in invert emulsion should demonstrate higher efficacy compared to the unformulated form of these strains. This means that the effective formulation should enhance the efficacy of formulated strains.
5. Details on successful examples demonstrating the higher efficacy of formulated strains of entomopathogenic fungi in invert emulsions versus the unformulated were given in the present article.
6. All methods and techniques adjusted and described in the present research are reproducible and give good results when applied properly.

## Additional information

### Background

An emulsion is a mixture in which two immiscible substances, like oil and water, stay mixed together with the help of a third substance called an emulsifier. An invert emulsion or “backwards” emulsion refers to an emulsion in which oil is the continuous or external phase and water is the dispersed or internal phase so that, in a given period of time after mixing oil and water phases with the help of an emulsifier, the emulsion may break down and may return gradually to the oil and water split. Therefore, there is a crucial need for improving the performance of these emulsions by developing effective techniques of mixing to increase the homogeneity of the emulsion and finding new and effective emulsifiers to increase the emulsion stability for long time.

Invert emulsions (water-in-oil type) are the most frequently used type of invert emulsions. They are mainly consisting of two liquid phases: dispersed phase (water) and continuous phase (oil). Emulsifiers that are introduced into these emulsions are usually included in the ingredients of the two phases before mixing them, they might be oil-soluble emulsifiers and water soluble emulsifiers. The water phase introduced into this emulsion is usually homogenized with oil phase at a high speed (20,000 rpm for 1.5 min using a homogenizer), thus the smaller water droplets in the final emulsion are completely surrounded by the larger oil droplets to produce water in oil emulsion. Therefore, during application, the oil droplets prevent the water droplets from evaporation especially at hot and dry conditions.

Invert emulsions (water-in-oil type) can be used for many purposes such as formulation of microorganisms, manufacturing of cosmetics and in food additives, in drilling fluid systems etc. [1], [2], [3], [4]. The formulation of microorganisms for being used as biocontrol agents of insect pests of crops, plant diseases and noxious weeds is one of the most important uses of these emulsions. Among the species of microorganisms that can be formulated in invert emulsion are the entomopathogenic fungi, and at hot dry conditions, water in the conidial suspension of these fungi based on water only will evaporate and dry up quickly after application but, if this water is incorporated into the invert emulsion as described above, it will not evaporate quickly following application due to the above-mentioned reason.

It is noteworthy to mention that the conventional method used for control of insect pests, plant diseases and weeds is the use of synthetic pesticides but the intensive use of these pesticides in crop protection provoked several ecological problems such as environmental pollution, toxicity hazards etc. One of the ecologically compatible alternative approach for using chemical pesticides is the use of antagonistic fungi as biocontrol agents of these pests. Example of these fungi is the use of entomopathogenic fungi which are specific for insects since their conidia can adhere to the insect cuticle then germinate and penetrate it. Following penetration, they can enter the cavity or haemocoel of attacked insects where they can grow and develop profusely to fill the inside of infected insects causing eventually their death. After death, the internal mycelium of these fungi emerges to outside then sporulates profusely on the outer surfaces of insect's cadaver giving new conidia that can disseminate everywhere and infect new healthy insects. In general, these fungi are characterized by having the ability to attack the adult insects and their developmental stages and then propagate intensively after successful host infection and distribution within the contaminated habitats (horizontal transmission). These characteristics make these fungi excellent candidates as biocontrol agents in the integrated management strategy of insects. Finally, it is important to indicate that the suggested formulation of invert emulsion has no environmental side-effects or harmful toxic effects since they are mainly composed of natural substances that are used as food additives or for manufacturing of cosmetics. Moreover, the applied fungal species introduced into these emulsions are easy to isolate in nature and easy to cultivate on culture medium, in addition to that they are cheap to maintain and easy to apply.

One important advantage of using invert emulsion in formulation of the entomopathogenic fungi is to enhance their efficacy during application against targeted insects since these emulsions contain the necessary water for germination of fungal conidia during or after application. Early studies conducted in our laboratory on testing the efficacy of formulated species of entomopathogenic fungi in invert emulsion have shown that the tested species like *Metarhizium anisoplia* and *Beauveria bassiana* have demonstrated higher efficacy compared to the unformulated forms of these species when tested against the following insect and pest species: tobacco whitefly (*Bemissia tabaci*), rice weevils (*Sitophilus oryzae*), granary weevils (*Sitophilus granarius*), lesser grain borers (*Rhyzopertha dominica*), and almond bark beetles (*Scolytus amygdali*), yellow mealworm (*Tenebrio molitor*) and red spidermites (*Tetranychus cinnabarinus*) [5], [6], [7], [8], [9], [10], [11].

## Figures and Tables

**Table 1:** Combinations of invert emulsion formulation (IE) based on variable percentage of ingredients of oil phase and water phase composing the emulsion.

Combination number (IE)	% of ingredients (w/w) of water phase and oil phase composing the formulation of invert emulsion (IE)							Stability of prepared emulsion	Viscosity of prepared emulsion
	Soybean oil	Coconut oil	Tween 20	Sterile distilled water	Conidial suspension of entomopathogenic fungi	Glycerin	Dehymuls k		
IE # 1	29.00	18.50	2.50	22.50	22.50	4.00	1.00	Less stable	Viscous
IE # 2	29.00	18.00	3.00	21.00	24.00	4.75	0.25	Less stable	Not viscous
IE # 3	28.50	19.00	2.50	22.00	23.00	4.50	0.50	Less stable	Not viscous
IE # 4	28.50	19.50	2.00	22.50	22.50	4.25	0.75	Stable for long time	Not viscous
IE # 5	28.50	19.00	2.50	23.00	22.00	4.00	1.00	Less stable	Viscous
IE # 6	29.00	18.00	3.00	23.00	22.00	4.00	1.00	Less stable	Viscous

**Table 2:** Mortality of adult insects of *Sitophilus granarius*, *Sitophilus oryzae* and *Rhyzopertha dominica* due to the treatment with unformulated and formulated *Metarhizium anisopliae* (strain MAPAL2) in invert emulsion. Incubation of the treated and control insects was done at 25 ± 1 °C for 10 days.

Treatments with *M. anisopliae* (strain MAPAL2) and control	Mean % of adult mortality in treated stored-grain insects with M. anisopliae*			Average mean % of adult mortality of insects/strain of entomopathogenic fungi**
	*Sitophilus granaries* (strain SGPAL)	*Sitophilus oryzae* (strain SOPAL)	*Rhyzopertha dominica* (strain RDPAL)	
Formulated *M. anisopliae* in invert emulsion	75.75 ± 3.19	77.5 ± 3.04	87.5 ± 3.04	80.25 ± 4.94c
Unformulated *M. anisopliae* (conidial suspension in water)	52.5 ± 4.55	62.5 ± 1.80	57.5 ± 1.10	57.50 ± 4.08b
Blank formulation of invert emulsion	12.5 ± 1.70	19.0 ± 2.74	25.0 ± 3.53	18.83 ± 5.10a
Untreated insects or the control treatment	10.0 ± 3.50	9.0 ± 2.24	7.5 ± 1.80	8.83 ± 1.03a

* Data presented here represent means (±SEM) of adult insects mortality of 4 replicates each. Each replicate represents a plastic pot with wheat grain (20 g) treated with unformulated or formulated *M. anisopliae* followed by introduction of 10 adult insects of each strain of stored-grain insects.

** Average of the mean mortality % of the three strains of stored-grain insects. The average means followed by the same letter are not significantly different at *P* = 0.05 using ANOVA and Duncan's Multiple Range test.

**Table 3:** Mortality of adult insects of *Sitophilus granarius*, *Sitophilus oryzae* and *Rhyzopertha dominica* due to the treatment with unformulated and formulated *Beauveria bassiana* (strain BBPAL1) in invert emulsion. Incubation of the treated and control insects was done at 25 ± 1 °C for 10 days.

Treatments with *B. bassiana* (strain BBPAL1) and control	Mean % of adult mortality in treated stored-grain insects with B. bassiana*			Average mean % of adult mortality of insects/strain of entomopathogenic fungi**
	*Sitophilus granaries* (strain SGPAL)	*Sitophilus oryzae* (strain SOPAL)	*Rhyzopertha dominica* (strain RDPAL)	
Formulated *B. bassiana* in invert emulsion	81.5 ± 2.06	85.5 ± 3.64	92.5 ± 4.33	86.50 ± 4.55c
Unformulated *B. bassiana* (conidial suspension in water)	59.0 ± 6.74	62.5 ± 5.59	65.0 ± 3.53	62.16 ± 2.46b
Blank formulation of invert emulsion	12.0 ± 1.89	16.5 ± 3.77	24.25 ± 3.77	17.58 ± 5.10a
Untreated insects or the control treatment	8.5 ± 2.50	10.5 ± 2.87	8.25 ± 3.42	9.08 ± 1.00a

* Data presented here represent means (±SEM) of adult insects mortality of 4 replicates each. Each replicate represents a plastic pot with wheat grain (20 g) treated with unformulated or formulated *B. bassiana* followed by introduction of 10 adult insects of each strain of stored-grain insects.

** Average of the mean mortality % of the three strains of stored-grain insects. The average means followed by the same letter are not significantly different at *P* = 0.05 using ANOVA and Duncan's Multiple Range test.

**Table 4:** Mortality of adult insects of *Scolytus amygdali* due to the treatment with unformulated and formulated *Beauveria bassiana* (strain 149) in invert emulsion. Incubation of the treated and control insects was done at 25 ± 2 °C for 4 and 10 days.

Treatments with *B. bassiana* (strain 149) and control	Mean % of adult mortality in treated almond bark beetles with B. bassiana*	
	4 days after the treatment	10 days after the treatment
Formulated *B. bassiana* in invert emulsion	43.3 ± 6.2c**	100 ± 5.2C**
Unformulated *B. bassiana* (conidial suspension in water)	16.7 ± 3.1b	60.0 ± 4.3b
Blank formulation of invert emulsion	10.0 ± 1.9a	16.7 ± 4.9a
Untreated insects or the control treatment	0 ± 0a	6.7 ± 2.7a

* Data presented here represent means (±SEM) of adult insects mortality of 4 replicates each. Each replicate represents one Petri dish with 10 males and 10 females of *S. amygdali* treated with one of the four treatments. Observed dead adult 4 days after the treatment have manifested no movement especially when excited with a needle prick whereas, observed dead adults 10 days after the treatment have manifested white mycelium growth typical to *B. bassiana* covering partially or completely the outer surfaces of dead adults.

** Means in the same column followed by the same letter are not significantly different (*P* < 0.05) using ANOVA and DMRT.

**Table 5:** Mortality of adult insects of *Rhyzopertha dominica* due to the treatment with unformulated and formulated *Metarhizium anisopliae* (strain Meta 1) in invert emulsion. Incubation of the treated and control insects was done at 23 ± 2 °C 75 ± 5% R.H. for 7 days.

Treatments with *M. anisopliae* (strain Meta 1) and control	Mean % of adult mortality in treated lesser grain borers with M. bassiana*
Formulated *M. anisopliae* in invert emulsion	92.0 ± 4.9c**
Unformulated *M. anisopliae* (conidial suspension in water)	53.3 ± 6.5b
Blank formulation of invert emulsion	25.3 ± 4.8a
Untreated insects or the control treatment	13.3 ± 2.8a

* Data presented here represent means (±SEM) of adult insects mortality of 4 replicates each. Each replicate represents a plastic pot with wheat grain (20 g) treated with unformulated or formulated *M. anisopliae* followed by introduction of 20 adult insects of each strain of stored-grain insects.

** Means of mortality % of the insects followed by the different letters are significantly different at *P* = 0.05 using ANOVA and Tukey-HSD test.

**Table 6:** Mortality of insect larvae of *Tenebrio molitor* due to the treatment with unformulated and formulated *Beauveria bassiana* (strain BG 1) in invert emulsion. Incubation of the treated and control insect larvae was done at 25 ± 1 °C for 7 days.

Treatments with *B. bassiana* (strain BG 1) and control	Mean % of larvae mortality in treated yellow mealworm with B. bassiana*
Formulated *B. bassiana* in invert emulsion	96.6 ± 5.7c**
Unformulated *B. bassiana* (conidial suspension in water)	40.0 ± 3.3b
Blank formulation of invert emulsion	23.3 ± 3.4b
Untreated insects or the control treatment	0 ± 0a

* Data presented here represent means (±SEM) of adult insects mortality of 4 replicates. Each replicate represents one Petri dish with wheat bran (20 g) treated with unformulated or formulated *B. bassiana* followed by introduction of 10 larvae of *T. molitor* into each petri dish.

** Means followed by the same letter are not significantly different (*P* < 0.05) using ANOVA and Tukey's HSD test.
